# Educational technologies to promote the sexual and reproductive health of women with HIV: an integrative review

**DOI:** 10.1590/0034-7167-2024-0079

**Published:** 2025-06-27

**Authors:** Karyanna Alves de Alencar Rocha, Daniel de Macedo Rocha, Marcela Antonini, Henrique Ciabotti Elias, Elucir Gir, Renata Karina Reis

**Affiliations:** I Universidade de São Paulo. Ribeirão Preto, São Paulo, Brazil

**Keywords:** Educational Technology, HIV, Reproductive Health, Sexual Health, Women, Tecnología Educacional, VIH, Salud Reproductiva, Salud Sexual, Mujeres

## Abstract

**Objectives:**

to map the scientific evidence on educational technologies developed for women with HIV/AIDS in relation to knowledge and behavior about sexual and reproductive health.

**Methods:**

integrative review, using databases such as MEDLINE, Web of Science, SCOPUS, EMBASE, LILACS and BDENF. Methodological quality was assessed using the Critical Appraisal Skills Program, and levels of evidence were determined using Fineout-Overholt. Primary studies were included and clinical trial protocols were excluded. The results were analyzed descriptively and qualitatively, resulting in two categories.

**Results:**

of the studies selected, four dealt with educational technologies related to sexual behavior and reproductive planning, while five addressed the puerperal pregnancy cycle. These studies were considered to have good methodological quality, low bias and proven effectiveness.

**Conclusions:**

the evidence synthesis highlighted the importance of disseminating knowledge about contraceptive and conception options to prevent avoidable complications in women with HIV of reproductive age.

## RESUMEN

**Objetivos:**

mapear pruebas científicas de tecnologías educativas para mujeres con VIH/SIDA sobre conocimientos y comportamientos en salud sexual y reproductiva.

**Métodos:**

se efectuó una revisión integradora en las bases de datos MEDLINE, Web of Science, SCOPUS, EMBASE, LILACS y BDENF. La calidad metodológica se evaluó con el Critical Appraisal Skills Program, y los niveles de evidencia se determinaron según Fineout-Overholt. Se incluyeron estudios primarios y se excluyeron protocolos de estudios clínicos. Los resultados se analizaron descriptiva y cualitativamente, dando lugar a dos categorías.

**Resultados:**

de los estudios seleccionados, cuatro trataban de tecnologías educativas relacionadas con el comportamiento sexual y la planificación reproductiva, mientras cinco abordaban el puerperio. Se consideró que estos estudios tenían buena calidad metodológica, escaso sesgo y eficacia demostrada.

**Conclusiones:**

la síntesis de las pruebas puso de relieve la importancia de difundir conocimientos sobre opciones anticonceptivas y conceptivas para prevenir complicaciones evitables en las mujeres con VIH en edad reproductiva.

## INTRODUCTION

Continuous involvement in HIV-related care is a global challenge^(1)^, especially for women of reproductive age, who are at high risk of abandoning care during pregnancy, childbirth and the postpartum period^(2)^. In 2022, around 39 million people were living with HIV, 53% of whom were women and girls. In that year, 46% of new infections occurred among women and girls, and 82% of pregnant women with HIV had access to antiretroviral drugs to prevent transmission to their children^(3)^.

Measures to prevent the sexual transmission of HIV in serodifferent couples, such as the use of Antiretroviral Therapy (ART) combined with the regular use of internal or external condoms, as well as behaviors that reduce exposure to HIV, increase the effectiveness of prevention and promote greater safety in the sexual and reproductive lives of women living with HIV and their partners^(4)^. However, these practices, which aim to expand conception and contraception strategies between couples with different or the same serology, require changes and adaptations in relationships and sexual behavior.

Educational interventions, both non-electronic such as support groups and behavioral counseling^(5)^, and through Educational Technologies (ET) and Mobile Health (mHealth)^(6,7)^, help women prevent the sexual transmission of HIV. ET and mHealth overcome barriers of access, cost, transportation and time, offering advantages over traditional interventions^(6)^. Used by health professionals, these technologies inform about transmission risks, promote appropriate communication between couples^(4)^ and improve adherence to health care, enhancing the quality of care in clinical practice^(8-10)^.

Seeking to promote preventive strategies to reduce HIV transmission, some studies have focused on condom use and adherence to drug treatment^(2,5)^. However, there are gaps in the literature on HT that seek to contribute to knowledge and behavior related to sexual and reproductive health, while at the same time providing information on effective contraceptive options that include reproductive planning, reception and counseling for women living with HIV/AIDS.

Given the above and the lack of protocols and reports of systematic reviews registered in the International Prospective Register of Systematic Reviews (PROSPERO), there was a need to discuss in the scientific literature the educational technologies developed to promote or strengthen the knowledge and behavior of women living with HIV/AIDS about sexual and reproductive health.

## OBJECTIVES

To map the scientific evidence on educational technologies developed for women living with HIV/AIDS on knowledge and behavior about sexual and reproductive health.

## METHODS

### Ethical aspects

The study followed the ethical standards established by national and international regulatory bodies. The ideas of the authors of the publications used have been duly credited and respected.

### Research design

This is an integrative literature review (IR)^(10)^, based on the theoretical framework proposed by Whittemore and Knaf^(11,12)^ and conducted in six stages: identification of the topic and structuring of the research question; sampling or literature search; selection and eligibility; extraction of data from the included studies; evaluation and interpretation of the results; and presentation of the review or synthesis of knowledge. It was made possible using an IR protocol^(13)^ developed specifically for this study.

### Elaboration of the research question

The guiding question was drawn up based on the domains of the PICo strategy^(12)^, which considered productions^(14,15)^ that focused on the population of women living with HIV/AIDS, on the phenomenon of interest, educational technologies, and on the context of sexual and reproductive health. Thus, this review was guided by the following question: “What are the contributions of educational technologies to promoting the sexual and reproductive health of women living with HIV/AIDS?”.

### Literature search and sampling

Two researchers (P1, P2) independently carried out a search for studies on educational technologies related to HIV/AIDS in the MEDLINE via PubMed, SCOPUS, Web of Science, Embase, LILACS and BDENF via BVS databases. Access was via the CAPES Journal Portal in an area with IP recognized by USP. There was no time limit for the selection of studies, which included manuscripts published until September 30, 2023. Disagreements were resolved by a third reviewer (P3), comparing the search results and verifying differences in the findings.

In order to prepare and operationalize the search strategy, we selected controlled and non-controlled descriptors indexed in the Medical Subject Headings (MeSH), Health Sciences Descriptors (DeCS) and Embase Thesaurus (emtree Terms) vocabularies and their respective synonyms, as follows: i) #1 Infected pregnant women (discordant couples, HIV-positive pregnant women, women living with HIV/AIDS, serodiscordant partners, women living with HIV and AIDS, serodiscordant relationships, maternal transmission of HIV); ii) #2 mobile health (m-health, Educational Technology, Educational Technologies, Instructional Technology, Instructional Technologies); iii) #3 Reproductive Health (Reproductive Health, Sexual Health, Sexual, Family Planning, Reproductive Desire, fertility desire, Fertility Intention). The strategy adopted took into account the specificities of each database consulted (Chart 1).

**Chart 1:** Search strategy generated after electronic consultation of the databases of interest, Ribeirão Preto, São Paulo, Brazil. 2024

Databases	Search strategies
**MEDLINE/Pubmed**	(((((((((Infected Pregnant Women) OR (discordant couples)) OR (HIV-positive pregnant women)) OR (women living with HIV/AIDS)) OR (Serodiscordant Partners)) OR (Women Living with HIV and AIDS)) OR (serodiscordant relationships)) OR (maternal transmission of HIV)) AND (((mobile health) OR (m-health)) OR (((((Educational Technology) OR (Educational Technologies)) OR (Educational Technologies)) OR (Instructional Technology)) OR (Instructional Technologies)))) AND ((((((((Reproductive Health) OR (Health, Reproductive)) OR (Sexual Health)) OR (Health, Sexual)) OR (Family Planning)) OR (Reproductive Desire)) OR (fertility desire)) OR (Fertility Intention))
**SCOPUS**	(( TITLE-ABS-KEY ( infected AND pregnant AND women ) OR TITLE-ABS-KEY ( discordant AND couples ) OR TITLE-ABS-KEY ( hiv-positive AND pregnant AND women ) OR TITLE-ABS-KEY ( women AND living AND with AND hiv/aids ) OR TITLE-ABS-KEY ( serodiscordant AND partners ) OR TITLE-ABS-KEY ( women AND living AND with AND hiv AND aids ) OR TITLE-ABS-KEY ( serodiscordant AND relationships ) OR TITLE-ABS-KEY ( maternal AND transmission AND of AND hiv ) ) ) AND ((TITLE-ABS-KEY ( mobile AND health ) OR TITLE-ABS-KEY ( m-health ) OR TITLE-ABS-KEY ( educational AND technology ) OR TITLE-ABS-KEY ( educational AND technologies ) OR TITLE-ABS-KEY ( educational AND technologies ) OR TITLE-ABS-KEY ( instructional AND technology ) OR TITLE-ABS-KEY ( instructional AND technologies ) ) ) AND ( ( TITLE-ABS-KEY ( reproductive AND health ) OR TITLE-ABS-KEY ( health, AND reproductive ) OR TITLE-ABS-KEY ( sexual AND health ) OR TITLE-ABS-KEY ( health, AND sexual ) OR TITLE-ABS-KEY ( family AND planning ) OR TITLE-ABS-KEY ( instructional AND technology ) OR TITLE-ABS-KEY ( reproductive AND desire ) OR TITLE-ABS-KEY ( fertility AND desire ) OR TITLE-ABS-KEY ( fertility AND intention)))
**WEB OF SCIENCE**	(ALL=(Infected Pregnant Women) OR ALL=(discordant couples) OR ALL=(HIV-positive pregnant women) OR ALL=(women living with HIV AIDS) OR ALL=(Serodiscordant Partners) OR ALL=(Women Living with HIV and AIDS) OR ALL=(serodiscordant relationships) OR ALL=(maternal transmission of HIV)) AND (ALL=(mobile health) OR ALL=(m-health) OR ALL=(Educational Technology) OR ALL=(Educational Technologies) OR ALL=(Educational Technologies) OR ALL=(Instructional Technology) OR ALL=(Instructional Technologies)) AND (ALL=(Reproductive Health) OR ALL=(Health, Reproductive) OR ALL=(Sexual Health) OR ALL=(Health, Sexual) OR ALL=(Family Planning) OR ALL=(Reproductive Desire) OR ALL=(fertility desire) OR ALL=(Fertility Intentio))
**Embase**	(‘infected pregnant women’ OR (infected AND pregnant AND (‘women’/exp OR women)) OR ‘discordant couples’:ti,ab,kw OR ‘hiv-positive pregnant women’:ti,ab,kw OR ‘women living with hiv aids’:ti,ab,kw OR ‘serodiscordant partners’:ti,ab,kw OR (‘women living with hiv’:ti,ab,kw AND aids:ti,ab,kw) OR ‘serodiscordant relationships’:ti,ab,kw OR ‘maternal transmission of hiv’:ti,ab,kw) AND (‘mobile health’/exp OR ‘mobile health’ OR ‘m health’:ti,ab,kw OR ‘educational technology’:ti,ab,kw OR ‘educational technologies’:ti,ab,kw OR ‘instructional technology’:ti,ab,kw OR ‘instructional technologies’:ti,ab,kw) AND (‘health, reproductive’ OR ((‘health,’/exp OR health,) AND reproductive) OR ‘sexual health’:ti,ab,kw OR ‘health, sexual’:ti,ab,kw OR ‘family planning’:ti,ab,kw OR ‘reproductive desire’:ti,ab,kw OR ‘fertility desire’:ti,ab,kw OR ‘fertility intentio’:ti,ab,kw)
**LILACS e BDENF** **via BVS**	(((*Grávidas infectadas pelo HIV*) OR (*casais discordantes*) OR (*Grávidas soropositivas*) OR (*Mulheres vivendo com HIV/AIDS*) OR (*Parceiros sorodiscordantes*) OR (*Mulheres vivendo com HIV e AIDS*) OR (*relações sorodiscordantes*) OR (*transmissão materna do HIV*)) OR ((Infected Pregnant Women HIV) OR (discordant couples) OR (HIV-positive pregnant women) OR (women living with HIV/AIDS) OR (Serodiscordant Partners) OR (Women Living with HIV and AIDS) OR (serodiscordant relationships) OR (maternal transmission of HIV)) OR ((*Mujeres embarazadas infectadas HIV*) OR (*parejas discordantes*) OR (*mujeres embarazadas VIH positivas*) OR (*mujeres que viven con VIH/SIDA*) OR (*Parejas serodiscordantes*) OR (*Mujeres que viven con VIH y SIDA*) OR (*relaciones serodiscordantes*) OR (*transmisión materna del VIH*))) AND (((*saúde móvel*) OR (*m-saúde*) OR (*Tecnologia Educacional*) OR (*Tecnologias Educacionais*) OR (*Tecnologias Educacionais*) OR (*Tecnologia Instrucional*) OR (*Tecnologias Instrucionais*)) OR ((mobile health) OR (m-health) OR (Educational Technology) OR (Educational Technologies) OR (Educational Technologies) OR (Instructional Technology) OR (Instructional Technologies)) OR ((*salud móvil*) OR (m-health) OR (*Tecnología educativa*) OR (*Tecnologías educativas*) OR (*Tecnologías educativas*) OR (*Tecnología educativa*) OR (*Tecnologías educativas*))) AND (((*Saúde Reprodutiva*) OR (*Saúde Reprodutiva*) OR (*Saúde Sexual*) OR (*Saúde Sexual*) OR (*Planejamento Familiar*) OR (*Desejo Reprodutivo*) OR (*desejo de fertilidade*) OR (*Intenção de Fertilidade*)) OR ((Reproductive Health) OR (Reproductive Health) OR (Sexual Health) OR (Sexual Health) OR (Family Planning) OR (Reproductive Desire) OR (Desire to Fertility) OR (Fertility Intent) OR (*Salud reproductiva*) OR (*Salud reproductiva*) OR (*Salud sexual*) OR (*Salud sexual*) OR (*Planificación familiar*) OR (*Deseo reproductivo*) OR (*Deseo de fertilidad*) OR (*Intención de fertilidad*)))

**Figure 1:**
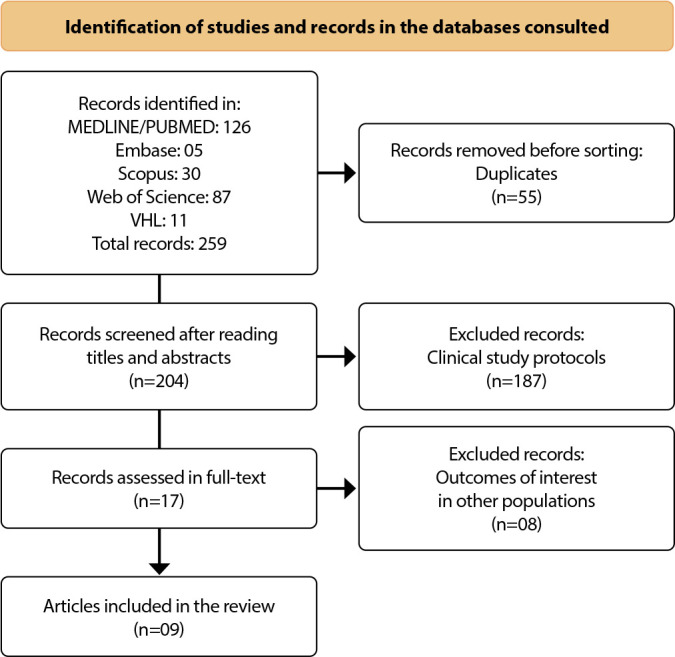
Process of selection, eligibility and inclusion of studies, Ribeirão Preto, São Paulo, Brazil, 2024

The route is described in figure 1, using the flowchart proposed by PRISMA (Preferred Reporting Items for Systematic Reviews and Meta-Analyses)^(16)^.

The files were exported to Rayyan software, where duplicate records were identified and excluded, as well as being selected independently by researchers P1 and P2. Initially, the titles and abstracts were read and the relevance test was applied, consisting of inclusion and exclusion criteria, which assessed the titles and abstracts and determined the potential for inclusion of the studies. All conflicts were managed by an independent third reviewer (P3) with experience in the field and in the research method. The concordance estimated in the selection process was estimated by the Concordance Index and totaled a score of 0.92. It should be noted that the sample composition was defined after full-text analysis of all the included references^(15)^.

This review included primary studies that developed, validated or evaluated the effects, contributions and impacts of TE on the sexual and reproductive health of women living with HIV/AIDS. To this end, TE was considered to be: videos, printed materials, educational games, software, booklets, software, theater, applications, programmed text messages, among others. With regard to sexual and reproductive health, the importance of a pleasurable and safe sex life was understood through terminology, and in the case of women, throughout the pregnancy-puerperium cycle, through information on sexuality, STI/AIDS prevention and the freedom to decide if they want to have children, when and how often they will have them, through access to information and contraceptive methods^(13,14)^.

### Categorization and evaluation of studies

Data extraction was also conducted independently by peers. A validated and adapted instrument was used^(17,18)^, including variables related to the identification of the studies, methodological aspects, ET developed, objective, limitations, main results and conclusions. The criteria proposed by the Critical Appraisal Skills Program (CASP)^(18)^ were used to assess methodological quality, and the seven-level classification proposed by Fineout-Overholt^(19)^ was used to measure the strength of the evidence.

The analysis of the evidence and the synthesis of the results were carried out in a descriptive and qualitative way of the themes of the articles, through a process of categorization, which resulted in the structuring of two categories according to the analysis of semantic similarity: Educational Technologies related to sexual behavior and reproductive planning; Educational Technologies related to the puerperal pregnancy cycle.

## RESULTS

Of the articles selected to make up the IR, four dealt with SRH related to sexual risk behavior and reproductive planning in women living with HIV/AIDS^(20-23)^ and five SRH related to the pregnancy-puerperium cycle of women living with HIV/AIDS^(24-28)^, all of which were published in English and in 7 different journals. As for the origin of the studies, they were carried out in countries with different levels of development, such as Kenya^(21,24,26,27)^, the United States^(20,28)^, South Africa^(22,25)^ and Brazil^(23)^. With regard to the year of publication, it can be seen that they were published in 2014^(24)^, 2017^(25)^, 2018^(20,26)^, 2019^(21)^, 2021^(22)^, 2022^(23)^ and 2023^(27,28)^. The predominant technology was the use of programmed text messages^(20,24-27)^, application^(21,28)^, software^(22)^, and educational booklet^(23)^.

As for methodological rigor, eight articles were classified as having good methodological quality and reduced bias (level A^(20,21,23-28)^), and level III evidence predominated^(21,22,24-27)^. The objectives of the studies were based on evaluating the effectiveness, applicability and acceptability of the proposed interventions, with generally positive results in all the studies.

Studies have highlighted factors that interfere with the sexual and reproductive health behavior of women living with HIV/AIDS, such as: adherence to ART during pregnancy or after childbirth, related to side effects^(20,21,26,28)^; concerns about safety regarding the disclosure of serological status^(25,26)^ lack of information about safe conception options among serodifferent couples^(20,21,23)^ distrust regarding risky behavior and sexual transmission^(23,25)^; possible risks in vertical transmission of the virus and infant prophylaxis^(26)^; concerns about fertility, efficacy of PrEP, STIs^(21)^; and postpartum safety^(27)^ (Chart 2).

**Chart 2:** Summary of primary studies related to the development of Educational Technologies on the sexual and reproductive health of women living with HIV/AIDS, 2024

Title	Year/ Country	Design/participants	Intervention	Objective	Results	NE^*^/ RM^†^
**Category 1: Educational technologies related to sexual behavior and reproductive planning**						
Mobile Health Intervention to Reduce HIV Transmission: A Randomized Trial of Behaviorally Enhanced HIV Treatment as Prevention (B-TasP)^(20)^	2018/ USA	Prospective cohort/ N= 383 men and n=117 women living with HIV.	Mobile Health intervention (scheduled text messages).	To test a behavioral intervention (mHealth) to improve medication adherence and reduce risk behaviors in People Living with HIV (PLHIV).	Participants showed greater adherence to ART, lower viral load, lower rates of substance use in the sexual context and greater endorsement of the beliefs of infectiousness and sexual transmission.	II/ A
A clinic-based tablet application to support safer conception among HIV serodiscordant couples in Kenya: feasibility and acceptability study^(21)^	2019/ Kenya	Intervention study/ N=74 serodifferent couples	SCIP-App text messaging application	To examine the feasibility and usability of short text messages in a mobile application to support safe conception in HIV-negative couples. The messages provided information on fertility and ovulation prediction, pre-exposure prophylaxis (PrEP), ART, condomless sex, STI diagnostic tests and treatment.	mHealth tools were considered acceptable for monitoring fertility indicators, promoting safe conception among couples and improving communication between health professional and patient.	III/ A
Positive Health Check intervention tool usage during a feasibility pilot in HIV primary care clinics^(22)^	2021/ South Africa	Intervention study/ N=97 PLHIV	Software-based counseling intervention.	Promote an internet-based intervention where users interact with a virtual doctor and, based on answers to personalized questions, the software offers content modules that address treatment initiation and adherence, reduction of sexual risk behavior, vertical transmission and injecting drug use.	The intervention involved the participants, aligning the topics with self-reported areas of interest, and was short enough for users to complete in a single consultation. The technology proved to be a promising tool.	III/ B
*Construção e validação de cartilha educacional sobre saúde sexual e reprodutiva para casais sorodiscordantes* ^(23)^	2022/ Brazil	Methodological study/ N=28 specialists in the field.	Educational booklet	To describe the process of constructing and validating an educational booklet on sexual and reproductive health for serodifferent couples.	All the items in the booklet had a percentage of agreement >80%, as did the overall average (91%). The content analysis showed significance (p>0.05) in all items and 100% agreement in the evaluation. The study concludes that the use of ET promotes individual empowerment and directs self-care.	IV/ A
**Category 2: Educational Technologies related to the puerperal pregnancy cycle**						
Texting improves testing a randomized trial of two-way SMS to increase postpartum prevention of mother-to-child transmission retention and infant HIV testing^(24)^	2014/ Kenya	Randomized clinical trial/ N=388 HIV+ women between 28 weeks gestation and delivery	Individually tailored, theory-based SMS (text messaging).	To determine whether interactive text messaging improved clinic attendance and early HIV testing rates in infants.	Women were significantly more likely to attend the clinic in the postpartum period, and the likelihood of infant HIV testing within 8 weeks was also higher. Text messaging significantly improved the frequency of maternal postpartum visits, but overall return rates for these visits remained low.	III/ A
Effectiveness of an SMS-based maternal mHealth intervention to improve clinical outcomes of HIV-positive pregnant women^(25)^	2017/ South Africa	Retrospective intervention study/ N=821 HIV+ pregnant women	SMS MAMA (text messages).	To evaluate the effectiveness of an mHealth intervention in prenatal care, to increase postnatal HIV testing in infants and improve postpartum outcomes in HIV+ women.	The intervention increased the number of women attending ANC appointments and vaginal deliveries. In addition to the first postpartum follow-up, it proved to be an effective intervention.	III/ A
SMS messaging to improve ART adherence: perspectives of pregnant HIV-infected women in Kenya on HIV-related message content^(26)^	2018/ Kenya	Focus group/ N=87 HIV+ pregnant women	Text messages (SMS)	Determine the appropriateness and preferred terminology of HIV-related content via text messaging to support HIV care.	The women expressed concerns about HIV-related privacy, and reported fear of having their HIV status revealed to people close to them. They also expressed a desire to receive text messages about ART side effects, vertical transmission and infant prophylaxis.	III/ A
The effect of an interactive weekly text-messaging intervention on retention in prevention of mother-to-child transmission of HIV care: a randomised controlled trial (WelTel PMTCT)^(27)^	2023/ Kenya	Randomized clinical trial/ N= 600 pregnant women, HIV+	WelTel PMTCT (text message intervention)	To investigate whether an intervention based on weekly text messages strengthens care to prevent mother-to-child transmission of HIV.	Text messaging was not directly associated with better retention in care for prevention of mother-to-child transmission and postpartum, or with linkage to health services within 30 months of delivery. It is believed that the increase in HIV-related health care is due to increased communication between health professional and user via cell phone.	III/ A
The Implementation of a GPS-Based Location-Tracking Smartphone App in South Africa to Improve Engagement in HIV Care: Randomized Controlled Trial^(28)^	2023/ USA	Randomized clinical trial/ N=200 pregnant women (≥28 weeks), HIV+	CareConekta (app)	To describe the feasibility, acceptability and initial effectiveness of using the CareConekta app to track the location of participants in order to help them find new facilities that offer ART if they travel > 50 km from the study site for > 7 days.	Lack of mobile data, difficulty installing the app and phone changes hindered the aim of the research with a GPS-based app. Acceptability measures were positive, but participants at follow-up showed a lack of understanding of the app’s purpose and function.	II/ A

* *LE - Level of evidence; †MR - Methodological rigor.*

With regard to the reasons for developing ET, the evidence points to women’s desire for greater access to information and guidance on safe reproductive planning and how to protect the baby^(24-27)^; contraceptive methods^(20,21,23)^; side effects of ART during pregnancy^(24,28)^; and the risk of sexual transmission of the infection^(20-23)^.

In addition, the use of ST with a focus on sexual and reproductive health can be associated with an increase in follow-up by health professionals and services^(20,22-26)^; conscious and safe sexual behavior among serodifferent couples^(20,23)^; a greater number of vaginal deliveries^(25)^; and control of viral load and reduction of vertical transmission^(26)^. In addition, the evidence highlights the potential use of mHealth as an increasingly accessible educational resource, promoting empowerment and directing the self-care and autonomy of women living with HIV^(20-28)^. The authors highlighted some limitations and risks of bias in the studies reviewed, including selection bias^(22-25,27)^, limited sample size^(20,21)^, occasional poor functioning of mobile ET^(26,27)^, data restricted to a single institution^(21)^ and results that cannot be generalized^(23)^.

## DISCUSSION

The recognition of sexual and reproductive health issues as a public health issue, driven by globalization, has led to the development of Educational Technologies (ET) for behavioral interventions. These technologies address risks in different contexts, STI/AIDS prevention and sexual and vertical transmission, as well as promoting reproductive planning and guiding care practices and clinical conduct. The study highlights the growing interest of researchers in the international literature in strengthening care and educational practices, guaranteeing sexual and reproductive rights for women with HIV/AIDS.

In this integrative review, evidence relevant to clinical nursing practice, such as randomized, observational and methodological clinical trials, shows that the predominant ST is based on text messages, with an emphasis on the use of smartphones and the internet. The analysis of the articles resulted in two categories: SRT related to risky sexual behavior and reproductive planning, and SRT related to the puerperal pregnancy cycle. This approach aims to support safe, effective and quality care in clinical nursing practice.

### Educational technologies related to sexual behavior and reproductive planning

The decentralized protocols for dealing with HIV have strengthened access to ART, but PLHIV have their comprehensive health needs not met, such as issues related to sexual and reproductive health^(21,24,25)^. Reproductive planning allows women living with HIV/AIDS to make reproductive and sexual behavior choices that have a positive impact on reproductive health, such as spacing pregnancies and using contraceptives^(23)^.

It is estimated that more than 80% of unplanned and/or unwanted pregnancies among these women are the result of sexual and reproductive health needs not being met effectively, highlighting a public health problem when related to unfavorable outcomes such as prematurity, late start of prenatal care, delayed access and low adherence to ART, increasing the risk of vertical transmission^(23)^.

The interventions found were effective in improving adherence to ART for both women and their partners, by focusing on cognitive-behavioral skills and strategies to resolve individual, situational and structural barriers^(20,21)^. Thus, cognitive-behavioral skills development techniques are also fundamental^(11)^ for more effective sexual health interventions.

The analysis of the types of CBT used to prevent sexual behavior and reproductive planning in women living with HIV/AIDS totaled four articles^(20-23)^, with little dissemination among the studies^(20)^. Of these, two^(20,21)^ were aimed at PLHIV including women, one at serodifferent couples^(22)^, and one^(23)^ by specialists in the field and also serodifferent couples.

In the publications analyzed, there was a predominance of hard ST, i.e. requiring technological resources, found in three of the studies^(20-22)^, focused on the following topics: prevention of sexual transmission of HIV, safe reproductive planning^(20,21)^, use of ART by the HIV-infected partner, Pre-Exposure Prophylaxis (PrEP) by the HIV-uninfected partner, condomless sex restricted to days with peak fertility, testing and treatment for STIs, voluntariness of medical male circumcision, vaginal self-insemination and assisted reproductive interventions. All the studies reflected the minimization of risk behaviour in serodifferent couples, while supporting pregnancy desires.

However, only one study^(21)^ highlighted the use of contraceptive methods as necessary information to be disseminated among women living with HIV/AIDS, albeit in a superficial way. This demonstrates the need to expand strategies that seek to guarantee sexual and reproductive rights, not only with regard to safe conception, but also to provide guidance on available and effective contraceptive options, which include reproductive planning, reception and counseling of women living with HIV/AIDS, based on scientific evidence.

In general, the results showed that the SMS developed significantly reduced HIV viral load^(20,22)^, increased adherence to ART^(20,21)^ and reduced new STI diagnoses^(21)^. In addition, four aspects were highlighted positively among the studies: ease of responding to SMS messages and incorporating the messages into daily life^(20,22-28)^; concerns around confidentiality and disclosure of information^(20-23)^; acceptability of mHealth tools to help with fertility monitoring^(20-22)^; and usefulness of mHealth tools to improve communication between couples and health professionals^(21,23)^.

The Educational Technologies (ET) analyzed include text messaging applications^(20,21,24-28)^, software^(22)^ and educational booklets^(23)^. These methods, in particular mHealth, facilitate the participation and involvement of participants by making information on the prevention of risky sexual behavior and reproductive planning more accessible. One study used an educational booklet to guide safe sexual and reproductive practices for people living with HIV (PLHIV) and their partners, promoting co-responsibility and autonomy in managing their own health.

### Educational technologies related to the pregnancy-puerperium cycle

The increased risk of HIV infection is also reported in pregnant women, who should be prioritized due to the historical, educational and structural contexts in which they live^(25)^. These conditions reflect the presence of obstacles, barriers and care challenges for tackling and controlling the HIV/AIDS epidemic not only in Brazil, but worldwide, and may explain the high prevalence of vertical transmission.

It is estimated that many HIV-positive pregnant women lose their follow-up care after giving birth^(24,25)^. It is currently recommended that HIV serological tests be carried out monthly for the first six months and at least bimonthly from the first year of life onwards for mothers and babies exposed to HIV^(30)^. This was one of the concerns addressed in the ST developed by the studies^(23,24,26)^.

In this category, six studies used ETE related to women living with HIV/AIDS during the puerperal pregnancy cycle^(24-28)^, including interventions based on text messages^(24-27)^, and apps^(28)^. Thus, there has been an effort to develop and disseminate electronic ETE aimed at HIV during the puerperal pregnancy cycle. This awakening to the use of information and communication ET is fundamental, as it has favored accessibility, the dissemination of knowledge, attractiveness and quick access to information through the studies presented^(24-28)^.

The development of ST using a methodological design such as randomized trials^(24-28)^ was identified in the analysis of the studies, demonstrating the high methodological rigor used in the development of ST for women living with HIV/AIDS during the pregnancy-puerperium cycle. Such studies are essential for health systems and nursing practice, due to their ability to reveal the cause and effect of different interventions^(29,30)^.

It was observed that access to cell phones was high among the studies^(24-28)^. Only a small proportion of the women who took part in the studies were excluded due to lack of access to a cell phone^(24-28)^. This coincides with other evidence available in the literature recommending greater use of available technologies to improve access and adherence to follow-up in health services and to ART^(31,32)^. It also contributes to an increase in the use of telecommunications as part of care and the creation of links between health professionals and the community^(24,33)^.

As public health strategies, when they are individually adapted they can be more effective than generic interventions^(34)^. A strength portrayed in one of the studies was the personalization of text messages based on the recipient’s gestational age, name, preferred time, desired language, delivery date and baby’s name. This made it possible to target messages to individuals, a feature that added a “personal touch”. Participants were also allowed to text, call or request a callback. This interactivity provided an opportunity to respond to participants’ needs and questions^(25)^.

In another study^(27)^, an approach was also taken in which participants who had disclosed their HIV status or did not have their own phone could choose to receive explicit HIV-related content. This approach offers a model for allowing intervention recipients to choose whether to receive open HIV-related SMS based on their individual preferences and situation, while also including protections against disclosure of HIV status.

Another aspect highlighted was the increase in the average number of antenatal and postnatal visits and follow-up^(24,28)^, as well as the fact that access to an available and mobile technology was associated with an increase in the number of vaginal births in one of the studies^(25)^. Based on these encouraging results, national maternal mHealth interventions should be seriously considered and evaluated. Carrying out an intervention to resemble real-life conditions, the long duration of follow-up, from the first antenatal visit to 30 months after delivery, was also another perceived strength^(27)^.

There is growing evidence that text message and mobile application-based approaches can improve adherence to care related to the pregnancy-postpartum cycle. However, there is no consensus in the field regarding the content of text messages, particularly the extent to which HIV can be openly addressed via SMS^(26,35,36)^.

Another ST for the promotion of HIV-related care during pregnancy was an app^(28)^, developed to track the location of participants in order to help them find health services that offer ART and prenatal care appointments in the event of travel. This was one of the first GPS-based mHealth studies, and although promising, it faced problems related to installation, operation and mobile internet access.

In nursing, health ETs are promising, attractive, innovative and effective methods for directing care and educational practices^(35)^, as they promote greater adherence to continuous care, welcoming, self-care, monitoring of risk conditions, evaluation of results and expansion of individual and collective knowledge, attitudes and skills.

However, most research focuses on identifying the epidemiological profile and measuring the impact of infection on different social segments^(36)^. Therefore, there are still gaps in the dissemination of knowledge about the development of ET, as well as its effects on access to care, health behavior and population information related to sexual health and HIV/AIDS.

### Study limitations

The main limitation of this Integrative Review is the possibility of missing relevant studies indexed in other databases. However, it was possible to map significant studies using the controlled descriptors and their respective synonyms, aligning them with the databases and research question. It is important to note that publication bias may be associated with the specific characteristics of each institution where the included studies were conducted, preventing the investigation of approaches in different contexts in the health care of women with HIV.

### Contributions to Nursing and Public Health

The results of this Integrative Review (IR) highlight that the inclusion of strategies involving educational technologies is innovative and has great potential to guide clinical nursing practice. The study represents an advance in the use of educational technologies, which are essential for reducing unprotected sex, promoting condom use, minimizing risk behaviors, reducing the number of partners and increasing adherence to HIV testing, vertical prevention and preand post-natal care. The role of nurses is crucial to ensure that these strategies are understood and applied correctly, maximizing their positive impact on public health.

These strategies contribute to the development of knowledge and behaviors related to reproductive planning, care, necessary interventions, good practices and comprehensive care on conception, contraception and HIV/AIDS. In these areas, nurses play a key role as facilitators of health education in health services, using their specialized knowledge to implement and adapt these educational technologies effectively.

## CONCLUSIONS

This review provided a relevant synthesis of the literature on the development of Educational Technologies (ET), highlighting software, applications, text messages and booklets. The studies showed the potential of these ETs in promoting knowledge and behavior in sexual health and reproductive planning, validating their effectiveness, sensitivity and precision in various contexts of nursing practice in women’s health. Interventions through smartphones and text messages were predominant, presenting remarkable potential to expand the reach of mHealth by overcoming physical barriers and mitigating stigmas.

These approaches provide access to information, support adherence to care and promote autonomy in a private and conscious manner, helping to improve reproductive planning, assistance, targeting of care and the formulation of comprehensive care.
