# Supplementary material for: Educational technologies to promote the sexual and reproductive health of women with HIV: an integrative review
Source: Rev Bras Enferm. 2025 Jun 27;78(2):e20240079. doi: 10.1590/0034-7167-2024-0079 (PMC12208682; doi:10.1590/0034-7167-2024-0079)
Supplement: Translation

# Translation: Tecnologias educacionais para promoção da saúde sexual e reprodutiva de mulheres com HIV: revisão integrativa

**DOI:** 10.1590/0034-7167-2024-0079pt

**Authors:** Karyanna Alves de Alencar Rocha, Daniel de Macedo Rocha, Marcela Antonini, Henrique Ciabotti Elias, Elucir Gir, Renata Karina Reis

**Affiliations:** I Universidade de São Paulo. Ribeirão Preto, São Paulo, Brasil

**Keywords:** Tecnologia Educacional, HIV, Saúde Reprodutiva, Saúde Sexual, Mulheres

## Abstract

**Objetivos:**

mapear as evidências científicas sobre as tecnologias educacionais desenvolvidas para mulheres com HIV/aids em relação ao conhecimento e ao comportamento sobre saúde sexual e reprodutiva.

**Métodos:**

revisão integrativa, utilizando bases de dados como MEDLINE, Web of Science, SCOPUS, EMBASE, LILACS e BDENF. A qualidade metodológica foi avaliada usando o *Critical Appraisal Skills Program*, e os níveis de evidência determinados mediante *Fineout-Overholt*. Foram incluídos estudos primários e excluídos os protocolos de estudos clínicos. Os resultados foram analisados de forma descritiva e qualitativa, resultando em duas categorias.

**Resultados:**

dos estudos selecionados, quatro trataram de tecnologias educacionais relacionadas ao comportamento sexual e planejamento reprodutivo, enquanto cinco abordaram o ciclo gravídico puerperal. Esses estudos foram considerados de boa qualidade metodológica, baixo viés e efetividade comprovada.

**Conclusões:**

A síntese das evidências destacou a importância da disseminação do conhecimento sobre opções contraceptivas e concepcionais para prevenir complicações evitáveis em mulheres com HIV em idade reprodutiva.

## INTRODUÇÃO

O envolvimento contínuo nos cuidados relacionados ao HIV é um desafio global^(1)^, especialmente para mulheres em idade reprodutiva, que têm alto risco de abandonar os cuidados durante a gestação, parto e pós-parto^(2)^. Em 2022, cerca de 39 milhões de pessoas viviam com HIV, sendo 53% mulheres e meninas. Nesse ano, 46% das novas infecções ocorreram entre mulheres e meninas, e 82% das grávidas com HIV tiveram acesso a medicamentos antirretrovirais para prevenir a transmissão para seus filhos^(3)^.

As medidas de prevenção da transmissão sexual do HIV em casais sorodiferentes, como o uso da Terapia Antirretroviral (TARV) combinada ao uso regular de preservativo interno ou externo, além de comportamentos que reduzem a exposição ao HIV, aumenta a eficácia da prevenção e promove maior segurança na vida sexual e reprodutiva das mulheres que convivem com o HIV e o seu parceiro^(4)^. No entanto, as práticas que visam a ampliação de estratégias de concepção e contracepção entre o casal com sorologia distinta ou igual requerem mudanças e adaptações no relacionamento e no comportamento sexual.

Intervenções educacionais, tanto as não eletrônicas como grupos de apoio e aconselhamento comportamental^(5)^, quanto por Tecnologias Educacionais (TE) e Saúde Móvel (*mHealth*)^(6,7)^, ajudam mulheres a prevenir a transmissão sexual do HIV. As TE e *mHealth* superam barreiras de acesso, custo, transporte e tempo, oferecendo vantagens sobre intervenções tradicionais^(6)^. Utilizadas por profissionais de saúde, essas tecnologias informam sobre riscos de transmissão, promovem comunicação adequada entre casais^(4)^ e melhoram a adesão aos cuidados de saúde, aprimorando a qualidade da assistência na prática clínica^(8-10)^.

Procurando promover estratégias preventivas para a redução da transmissão do HIV, alguns estudos têm enfocado no uso do preservativo e na adesão ao tratamento medicamentoso^(2,5)^. No entanto, há lacunas na literatura sobre TE que busquem contribuir com o conhecimento e comportamento relacionados à saúde sexual e reprodutiva, e ao mesmo tempo, fornece informações sobre opções contraceptivas efetivas e que contemplem o planejamento reprodutivo, acolhimento e aconselhamento de mulheres que convivem com HIV/aids.

Diante do exposto e da ausência de protocolos e relatórios de revisões sistemáticas cadastradas no *International Prospective Register of Systematic Reviews* (PROSPERO), emergiu a necessidade de discutir na literatura científica as tecnologias educacionais desenvolvidas para promoção ou fortalecimento do conhecimento e comportamento de mulheres que convivem com HIV/aids acerca da saúde sexual e reprodutiva.

## OBJETIVOS

Mapear as evidências científicas sobre as tecnologias educacionais desenvolvidas para mulheres que convivem com HIV/aids acerca do conhecimento e comportamento sobre a saúde sexual e reprodutiva.

## MÉTODOS

### Aspectos éticos

O estudo seguiu as normas éticas estabelecidas pelos órgãos reguladores nacionais e internacionais. As ideias dos autores das publicações utilizadas foram devidamente creditadas e respeitadas.

### Delineamento da pesquisa

Trata-se de uma revisão integrativa da literatura (RI)^(10)^, fundamentada no referencial teórico proposto por Whittemore e Knaf^(11,12)^ e conduzida em seis etapas: identificação do tema e estruturação da questão de pesquisa; amostragem ou busca na literatura; seleção e elegibilidade; extração de dados dos estudos incluídos; avaliação e interpretação dos resultados; e apresentação da revisão ou síntese do conhecimento. Foi viabilizada a partir de um protocolo^(13)^ de RI desenvolvido especificamente para este estudo.

### Elaboração da questão de pesquisa

A questão norteadora foi elaborada a partir dos domínios da estratégia PICo^(12)^, em que foram consideradas as produções^(14,15)^ que se concentraram na população das mulheres que convivem o HIV/aids, no fenômeno de interesse as TE e no contexto a saúde sexual e reprodutiva. Assim, esta revisão foi conduzida pela seguinte pergunta: “Quais as contribuições das tecnologias educacionais para a promoção da saúde sexual e reprodutiva mulheres que convivem com HIV/aids?”.

### Busca na literatura e amostragem

Dois pesquisadores (P1, P2) realizaram, independentemente, uma busca de estudos sobre tecnologias educacionais relacionadas ao HIV/aids nas bases de dados MEDLINE via PubMed, SCOPUS, *Web of Science*, Embase, LILACS e BDENF via BVS. O acesso foi feito pelo Portal de Periódicos da CAPES em área com IP reconhecido pela USP. Não houve limite temporal para a seleção dos estudos, que incluíram manuscritos publicados até 30 de setembro de 2023. Discordâncias foram resolvidas por um terceiro revisor (P3), comparando os resultados das buscas e verificando diferenças nos achados.

Para a elaboração e operacionalização da estratégia de busca, selecionou-se descritores controlados e não controlados indexados nos vocabulários *Medical Subject Headings* (MeSH), Descritores em Ciências da Saúde (DeCS) e *Embase Thesaurus* (*emtree Terms*) e suas respectivas sinonímias, conforme segue: i) #1 *Infected Pregnant Women* (*discordant couples, HIV*-*positive pregnant women, women living with HIV/AIDS, Serodiscordant Partners, Women Living with HIV and AIDS, serodiscordant relationships, maternal transmission of HIV*); ii) #2 *mobile health* (*m-health, Educational Technology, Educational Technologies, Educational Technologies, Instructional Technology, Instructional Technologies*); iii) #3 *Reproductive Health* (*Health Reproductive, Sexual Health, Health, Sexual, Family Planning, Reproductive Desire, fertility desire, Fertility Intention*). A estratégia adotada considerou as especificidades de cada base consultada (Quadro 1).

**Quadro 1:** Estratégia de busca gerada após consulta eletrônica nas bases de interesse, Ribeirão Preto, São Paulo, Brasil, 2024

Bases de dados	Estratégias de busca
**MEDLINE/Pubmed**	(((((((((*Infected Pregnant Women*) *OR* (*discordant couples*)) *OR* (*HIV-positive pregnant women*)) *OR* (*women living with HIV/AIDS*)) *OR* (*Serodiscordant Partners*)) *OR* (*Women Living with HIV and AIDS*)) *OR* (*serodiscordant relationships*)) *OR* (*maternal transmission of HIV*)) *AND* (((*mobile health*) *OR* (*m-health*)) *OR* (((((*Educational Technology*) *OR* (*Educational Technologies*)) *OR* (*Educational Technologies*)) *OR* (*Instructional Technology*)) *OR* (*Instructional Technologies*)))) *AND* ((((((((*Reproductive Health*) *OR* (*Health, Reproductive*)) *OR* (*Sexual Health*)) *OR* (*Health, Sexual*)) *OR* (*Family Planning*)) *OR* (*Reproductive Desire*)) *OR* (*fertility desire*)) *OR* (*Fertility Intention*))
**SCOPUS**	(( *TITLE-ABS-KEY* ( *infected AND pregnant AND women* ) *OR TITLE-ABS-KEY* ( *discordant AND couples* ) *OR TITLE-ABS-KEY* ( *hiv-positive AND pregnant AND women* ) *OR TITLE-ABS-KEY* ( *women AND living AND with AND hiv/aids* ) *OR TITLE-ABS-KEY* ( *serodiscordant AND partners* ) *OR TITLE-ABS-KEY* ( *women AND living AND with AND hiv AND aids* ) *OR TITLE-ABS-KEY* ( *serodiscordant AND relationships* ) *OR TITLE-ABS-KEY* ( *maternal AND transmission AND of AND hiv* ) ) ) *AND* ((*TITLE-ABS-KEY* ( *mobile AND health* ) *OR TITLE-ABS-KEY* ( *m-health* ) *OR TITLE-ABS-KEY* ( *educational AND technology* ) *OR TITLE-ABS-KEY* ( *educational AND technologies* ) *OR TITLE-ABS-KEY* ( *educational AND technologies* ) *OR TITLE-ABS-KEY* ( *instructional AND technology* ) *OR TITLE-ABS-KEY* ( *instructional AND technologies* ) ) ) *AND* ( ( *TITLE-ABS-KEY* ( *reproductive AND health* ) *OR TITLE-ABS-KEY* ( *health, AND reproductive* ) *OR TITLE-ABS-KEY* ( *sexual AND health* ) *OR TITLE-ABS-KEY* ( *health, AND sexual* ) *OR TITLE-ABS-KEY* ( *family AND planning* ) *OR TITLE-ABS-KEY* ( *instructional AND technology* ) *OR TITLE-ABS-KEY* ( *reproductive AND desire* ) *OR TITLE-ABS-KEY* ( *fertility AND desire* ) *OR TITLE-ABS-KEY* ( *fertility AND intention*)))
***WEB OF SCIENCE***	(*ALL*=(*Infected Pregnant Women*) *OR ALL*=(*discordant couples*) *OR ALL*=(*HIV-positive pregnant women*) *OR ALL*=(*women living with HIV AIDS*) *OR ALL*=(*Serodiscordant Partners*) *OR ALL*=(*Women Living with HIV and AIDS*) *OR ALL*=(*serodiscordant relationships*) *OR ALL*=(*maternal transmission of HIV*)) *AND* (*ALL*=(*mobile health*) *OR ALL*=(*m-health*) *OR ALL*=(*Educational Technology*) *OR ALL*=(*Educational Technologies*) *OR ALL*=(*Educational Technologies*) *OR ALL*=(*Instructional Technology*) *OR ALL*=(*Instructional Technologies*)) *AND* (*ALL*=(*Reproductive Health*) *OR ALL*=(*Health, Reproductive*) *OR ALL*=(*Sexual Health*) *OR ALL*=(*Health, Sexual*) *OR ALL*=(*Family Planning*) *OR ALL*=(*Reproductive Desire*) *OR ALL*=(*fertility desire*) *OR ALL*=(*Fertility Intentio*))
**Embase**	(*‘infected pregnant women’ OR* (*infected AND pregnant AND* (*‘women’*/*exp OR women*)) *OR ‘discordant couples’:*ti,ab,kw *OR ‘hiv-positive pregnant women’:*ti,ab,kw *OR ‘women living with hiv aids’:*ti,ab,kw *OR ‘serodiscordant partners’:*ti,ab,kw *OR* (*‘women living with hiv’:*ti,ab,kw *AND aids:*ti,ab,kw) *OR ‘serodiscordant relationships’:*ti,ab,kw *OR ‘maternal transmission of hiv’:*ti,ab,kw) *AND* (*‘mobile health’*/*exp OR ‘mobile health’ OR ‘m health’:*ti,ab,kw *OR ‘educational technology’:*ti,ab,kw *OR ‘educational technologies’:*ti,ab,kw *OR ‘instructional technology’:*ti,ab,kw *OR ‘instructional technologies’:*ti,ab,kw) *AND* (*‘health, reproductive’ OR* ((*‘health*,’/*exp OR health*,) *AND reproductive*) *OR ‘sexual health’:*ti,ab,kw *OR ‘health, sexual’:*ti,ab,kw *OR ‘family planning’:*ti,ab,kw *OR ‘reproductive desire’:*ti,ab,kw *OR ‘fertility desire’:*ti,ab,kw *OR ‘fertility intentio’:*ti,ab,kw)
**LILACS e BDENF via BVS**	(((Grávidas infectadas pelo HIV) *OR* (casais discordantes) *OR* (Grávidas soropositivas) *OR* (Mulheres vivendo com HIV/AIDS) *OR* (Parceiros sorodiscordantes) *OR* (Mulheres vivendo com HIV e AIDS) *OR* (relações sorodiscordantes) *OR* (transmissão materna do HIV)) *OR* ((*Infected Pregnant Women HIV*) *OR* (*discordant couples*) *OR* (*HIV-positive pregnant women*) *OR* (*women living with HIV/AIDS*) *OR* (*Serodiscordant Partners*) *OR* (*Women Living with HIV and AIDS*) *OR* (*serodiscordant relationships*) *OR* (*maternal transmission of HIV*)) *OR* ((*Mujeres embarazadas infectadas HIV*) *OR* (*parejas discordantes*) *OR* (*mujeres embarazadas VIH positivas*) *OR* (*mujeres que viven con VIH/SIDA*) *OR* (*Parejas serodiscordantes*) *OR* (*Mujeres que viven con VIH y SIDA*) *OR* (*relaciones serodiscordantes*) *OR* (*transmisión materna del VIH*))) *AND* (((saúde móvel) *OR* (m-saúde) *OR* (Tecnologia Educacional) *OR* (Tecnologias Educacionais) *OR* (Tecnologias Educacionais) *OR* (Tecnologia Instrucional) *OR* (Tecnologias Instrucionais)) *OR* ((*mobile health*) *OR* (*m-health*) *OR* (*Educational Technology*) *OR* (*Educational Technologies*) *OR* (*Educational Technologies*) *OR* (*Instructional Technology*) *OR* (*Instructional Technologies*)) *OR* ((*salud móvil*) *OR* (*m-health*) *OR* (*Tecnología educativa*) *OR* (*Tecnologías educativas*) *OR* (*Tecnologías educativas*) *OR* (*Tecnología educativa*) *OR* (*Tecnologías educativas*))) *AND* (((Saúde Reprodutiva) *OR* (Saúde Reprodutiva) *OR* (Saúde Sexual) *OR* (Saúde Sexual) *OR* (Planejamento Familiar) *OR* (Desejo Reprodutivo) *OR* (desejo de fertilidade) *OR* (Intenção de Fertilidade)) *OR* ((*Reproductive Health*) *OR* (*Reproductive Health*) *OR* (*Sexual Health*) *OR* (*Sexual Health*) *OR* (*Family Planning*) *OR* (*Reproductive Desire*) *OR* (*Desire to Fertility*) *OR* (*Fertility Intent*) *OR* (*Salud reproductiva*) *OR* (*Salud reproductiva*) *OR* (*Salud sexual*) *OR* (*Salud sexual*) *OR* (*Planificación familiar*) *OR* (*Deseo reproductivo*) *OR* (*Deseo de fertilidad*) *OR* (*Intención de fertilidad*)))

O percurso está descrito na figura 1, por meio do fluxograma proposto pelo PRISMA (*Preferred Reporting Items for Systematic Reviews and Meta-Analyses*)^(16)^.

**Figura 1:** Processo de seleção, elegibilidade e inclusão de estudos, Ribeirão Preto, São Paulo, Brasil, 2024

Os arquivos foram exportados para o *software Rayyan*, onde foi realizada a identificação e exclusão dos registros duplicados, assim como a seleção de forma independente entre os pesquisadores P1 e P2. Inicialmente, ocorreu a leitura dos títulos, resumos e aplicação do teste de relevância, composto por critérios de inclusão e exclusão, os quais avaliaram os títulos e resumos, e determinaram o potencial de inclusão dos estudos. Todos os conflitos foram gerenciados por um terceiro revisor (P3) independente, com experiência na área e no método de investigação. A concordância estimada no processo de seleção foi estimada pelo Índice de Concordância e totalizou escore de 0,92. Destaca-se que a composição amostral foi definida após análise de texto completo de todas as referências incluídas^(15)^.

Esta revisão incluiu estudos primários que desenvolveram, validaram ou avaliaram os efeitos, contribuições e impactos das TE na saúde sexual e reprodutiva de mulheres que convivem com o HIV/aids. Para tanto, considerou-se como TE: vídeos, impressos, jogos educativos, *softwares*, cartilhas, *softwares*, teatro, aplicativos, mensagens de texto programadas, entre outros. No tocante à saúde sexual e reprodutiva, compreendeu-se mediante terminologia, a importância de uma vida sexual prazerosa e segura, e no caso das mulheres, em todo o ciclo gravídico puerperal, através de informações sobre a sexualidade, prevenção de IST/aids e a liberdade para decidirem se querem ter filhos, quando e com que frequência irão tê-los, através do acesso à informação e aos métodos contraceptivos^(13,14)^.

### Categorização e avaliação dos estudos

A extração de dados também foi conduzida por pares, de forma independente. Utilizou-se um instrumento validado e adaptado^(17,18)^, sendo contempladas variáveis relacionadas à identificação dos estudos, aspectos metodológicos, TE desenvolvida, objetivo, limitações, principais resultados e conclusões. Para avaliar a qualidade metodológica considerou-se os critérios propostos pelo *Critical Appraisal Skills Programm* (CASP)^(18)^ e, para mensurar a força da evidência, utilizou-se a classificação dos setes níveis propostos por Fineout-Overholt^(19)^.

A análise das evidências e a síntese dos resultados foram realizadas de forma descritiva e qualitativa dos temas dos artigos, por meio de um processo de categorização, o qual resultou na estruturação de duas categorias conforme análise da similaridade semântica: Tecnologias Educacionais relacionadas ao comportamento sexual e planejamento reprodutivo; Tecnologias Educacionais relacionadas ao ciclo gravídico puerperal.

## RESULTADOS

Dos artigos selecionados para compor a RI, quatro abordavam sobre TE relacionadas ao comportamento sexual de risco e planejamento reprodutivo em mulheres que convivem com HIV/aids^(20-23)^ e cinco TE relacionadas ao ciclo gravídico puerperal de mulheres que convivem com HIV/aids^(24-28)^, sendo todos os estudos publicados no idioma inglês e publicados em 07 periódicos diferentes. Quanto à origem dos estudos, foram realizados em países com diferentes níveis de desenvolvimento, tais como o Quênia^(21,24,26,27)^, Estados Unidos^(20,28)^, África do sul^(22,25)^ e Brasil^(23)^. Em relação ao ano das publicações, observa-se que foram publicados em 2014^(24)^, 2017^(25)^, 2018^(20,26)^, 2019^(21)^, 2021^(22)^, 2022^(23)^ e 2023^(27,28)^. A tecnologia predominante foi o uso de mensagens de texto programadas^(20,24-27)^, aplicativos^(21,28)^, *softwares* ^(22)^, e cartilhas educacionais^(23)^.

Quanto ao rigor metodológico, oito artigos foram classificados como boa qualidade metodológica e viés reduzido (nível A^(20,21,23-28)^), e predominou o nível III de evidência^(21,22,24-27)^. Os objetivos dos estudos foram pautados na avaliação da eficácia, aplicabilidade e aceitabilidade das intervenções propostas, apresentando resultados positivos de modo geral em todos os estudos.

Os estudos evidenciaram os fatores que interferem no comportamento na saúde sexual e reprodutivas das mulheres que convivem com o HIV/aids, como: adesão à TARV durante a gravidez ou após o parto, relacionadas aos efeitos colaterais^(20,21,26,28)^; preocupações sobre a segurança quanto à revelação do status sorológico^(25,26)^ falta de informações sobre opções de concepção segura entre casais sorodiferentes^(20,21,23)^ desconfiança em relação ao comportamento de risco e transmissão sexual^(23,25)^; eventuais riscos na transmissão vertical do vírus e profilaxia infantil^(26)^; preocupação sobre fertilidade, eficácia da PrEP, IST^(21)^; e segurança no pós parto^(27)^ (Quadro 2).

**Quadro 2:** Síntese de estudos primários relacionados ao desenvolvimento de Tecnologias Educacionais acerca da saúde sexual e reprodutiva de mulheres que convivem com o HIV/aids, 2024

Título	Ano/ País	Delineamento/ participantes	Intervenção	Objetivo	Desfechos	NE^*^/ RM^†^
**Categoria 1: Tecnologias Educacionais relacionadas ao comportamento sexual e planejamento reprodutivo**						
*Mobile Health Intervention to Reduce HIV Transmission: A Randomized Trial of Behaviorally Enhanced HIV Treatment as Prevention (B-TasP)* ^(20)^	2018/ EUA	Coorte prospectivo/ N= 383 homens e n=117 mulheres vivendo com HIV.	Mobile Health da intervenção (mensagens de texto programadas).	Testar uma intervenção comportamental (mHealth) para melhorar a adesão à medicação e reduzir comportamentos de risco em Pessoas vivendo com HIV (PVHIV).	Os participantes demonstraram maior adesão à TARV, menor carga viral, taxas mais baixas de uso de substâncias no contexto sexual e maior endosso às crenças de infecciosidade e transmissão sexual.	II/ A
*A clinic-based tablet application to support safer conception among HIV serodiscordant couples in Kenya: feasibility and acceptability study* ^(21)^	2019/ Quênia	Estudo de intervenção/ N=74 casais sorodiferentes	Aplicativo de mensagens de texto SCIP-App	Examinar a viabilidade e usabilidade de mensagens de texto curtas em aplicativo móvel para apoiar concepção segura em casais sorodiferentes. As mensagens informavam sobre fertilidade e previsão de ovulação, profilaxia pré-exposição (PrEP), TARV, sexo sem preservativo, testes de diagnóstico e tratamento de IST.	As ferramentas *mHealth* foram consideradas aceitáveis ​​para monitorizar indicadores de fertilidade, promover uma concepção segura entre os casais e melhorar a comunicação entre profissional da saúde e paciente.	III/ A
*Positive Health Check intervention tool usage during a feasibility pilot in HIV primary care clinics* ^(22)^	2021/ África do sul	Estudo de intervenção/ N=97 PVHIV	Intervenção de aconselhamento baseada em software.	Promover uma intervenção baseada na internet, onde os usuários interagem com um médico virtual, e, com base nas respostas a perguntas personalizadas, o *software* oferece módulos de conteúdo que abordam o início e adesão ao tratamento, redução do comportamento de risco sexual, transmissão vertical e uso de drogas injetáveis.	A intervenção envolveu os participantes, alinhando os tópicos com as áreas de interesse autoindicadas e foram breves para os utilizadores concluírem numa única consulta. A tecnologia demonstrou consistir em uma ferramenta promissora.	III/ B
Construção e validação de cartilha educacional sobre saúde sexual e reprodutiva para casais sorodiscordantes^(23)^	2022/ Brasil	Estudo metodológico/ N=28 especialistas na área.	Cartilha educacional	Descrever o processo de construção e validação de uma cartilha educacional sobre saúde sexual e reprodutiva de casais sorodiferentes.	Todos os itens da cartilha obtiveram percentual de concordância > 80%, assim como na média global (91%). Na análise de conteúdo, foi verificada significância (p>0,05) em todos os itens e 100% de concordância na avaliação. O estudo conclui que o uso de TE promove empoderamento do indivíduo e direciona o autocuidado.	IV/ A
**Categoria 2: Tecnologias Educacionais relacionadas ao ciclo gravídico puerperal**						
*Texting improves testing a randomized trial of two-way SMS to increase postpartum prevention of mother-to-child transmission retention and infant HIV testing* ^(24)^	2014/ Quênia	Ensaio clínico randomizado/ N=388 mulheres HIV+ entre 28 semanas de gestação e o parto	SMS adaptada individualmente e baseada em teoria (mensagens de texto).	Determinar se as mensagens de texto interativas melhoraram as taxas de frequência clínica e de testes precoces de HIV em bebês.	As mulheres tiveram uma probabilidade significativamente maior de comparecer à clínica no pós-parto, e como também, a probabilidade de teste de HIV infantil dentro de 8 semanas foi maior. As mensagens de texto melhoraram significativamente a frequência das consultas maternas pós-parto, mas as taxas globais de retorno destas consultas permaneceram baixas.	III/ A
*Effectiveness of an SMS-based maternal mHealth intervention to improve clinical outcomes of HIV-positive pregnant women* ^(25)^	2017/ África do sul	Estudo de intervenção retrospectivo/ N=821 mulheres grávidas HIV+	SMS MAMA (mensagens de texto).	Avaliar a efetividade de uma intervenção *mHealth* nos cuidados durante o pré-natal, aumentar a testagem pós-natal de HIV em bebês e melhorar os desfechos pós-parto de mulheres HIV+.	A intervenção aumentou o número de mulheres atendidas nas consultas de CPN e partos vaginais. Além da realização do primeiro acompanhamento pós-parto, mostrando ser uma intervenção eficaz.	III/ A
*SMS messaging to improve ART adherence: perspectives of pregnant HIV-infected women in Kenya on HIV-related message content* ^(26)^	2018/ Quênia	Grupo focal/ N=87 mulheres grávidas HIV+	Mensagens de texto (SMS)	Determinar a conveniência e a terminologia preferida do conteúdo relacionado com o HIV através de mensagens de texto para apoiar os cuidados de HIV.	As mulheres expressaram preocupações à privacidade relacionada ao HIV, relataram medo de ter o seu estado sorológico revelado para pessoas próximas. Ademais, expressaram desejabilidade em receber SMS sobre efeitos secundários da TARV, modo de transmissão vertical e profilaxia infantil.	III/ A
*The effect of an interactive weekly text-messaging intervention on retention in prevention of mother-to-child transmission of HIV care: a randomised controlled trial (WelTel PMTCT)* ^(27)^	2023/ Quênia	Ensaio clínico randomizado/ N= 600 mulheres grávidas, HIV+	WelTel PMTCT (intervenção de mensagens de texto)	Investigar se uma intervenção baseada em mensagens de texto semanais fortalece os cuidados para prevenção da transmissão vertical do HIV para os bebês e pós-parto.	As mensagens de texto não foram diretamente associadas a uma melhor retenção nos cuidados de prevenção da transmissão vertical e pós-parto, ou à vinculação aos serviços de saúde em até 30 meses após o parto. Acredita-se que o aumento dos cuidados em saúde relacionados ao HIV se deve ao aumento da comunicação entre profissional da saúde e usuário via telefone celular.	III/ A
*The Implementation of a GPS-Based Location-Tracking Smartphone App in South Africa to Improve Engagement in HIV Care: Randomized Controlled Trial* ^(28)^	2023/ EUA	Ensaio clínico randomizado/ N=200 mulheres grávidas (≥28 semanas), HIV+	CareConekta (aplicativo)	Descrever a viabilidade, aceitabilidade e eficácia inicial do uso do aplicativo CareConekta para rastreio da localização das participantes com a finalidade de auxiliá-las a procurarem novas instalações que ofertam TARV em caso de viagem > 50 km do local do estudo por > 7 dias.	A falta de dados móveis, dificuldade na instalação do aplicativo e trocas de telefone e atrapalharam o objetivo da pesquisa com um aplicativo baseado em GPS. As medidas de aceitabilidade foram positivas, porém, os participantes no acompanhamento demonstraram falta de compreensão do propósito e da função do aplicativo.	II/ A

* *NE - Nível de evidência.; ^†^RM - Rigor metodológico.*

Com relação às razões para o desenvolvimento das TE, as evidências apontam para o desejo das mulheres em maior acessibilidade às informações e orientações sobre a realização de um planejamento reprodutivo seguro e como proteger o bebê^(24-27)^; métodos contraceptivos^(20,21,23)^; efeitos colaterais da TARV durante a gestação^(24,28)^; e risco de transmissão sexual da infecção^(20-23)^.

Ademais, o uso de TE com foco na saúde sexual e reprodutiva pode ser associado à um aumento do acompanhamento por parte dos profissionais e serviços de saúde^(20,22-26)^; comportamento sexual consciente e seguro entre casais sorodiferentes^(20,23)^; maior número de partos vaginais^(25)^; e controle da carga viral e redução da transmissão vertical^(26)^. Além disso, as evidências destacam o potencial uso da *mHealth* como recurso educacional cada vez mais acessível, promovendo o empoderamento e direcionando o autocuidado e autonomia das mulheres convivendo com o HIV^(20-28)^. Os autores destacaram algumas limitações e riscos de viés nos estudos revisados, incluindo viés de seleção^(22-25,27)^, tamanho amostral limitado^(20,21)^, funcionamento ocasionalmente deficiente de TE móveis^(26,27)^, dados restritos a uma única instituição^(21)^ e resultados que não podem ser generalizados^(23)^.

## DISCUSSÃO

O reconhecimento de questões relacionadas à saúde sexual e reprodutiva como uma vertente de saúde pública, impulsionado pela globalização, levou ao desenvolvimento de Tecnologias Educacionais (TE) para intervenções comportamentais. Essas tecnologias abordam riscos em diferentes contextos, prevenção de IST/AIDS e transmissão sexual e vertical, além de promover o planejamento reprodutivo e orientar práticas assistenciais e condutas clínicas. O estudo destaca o crescente interesse de pesquisadores na literatura internacional em fortalecer práticas assistenciais e educacionais, garantindo direitos sexuais e reprodutivos para mulheres com HIV/AIDS.

Nesta revisão integrativa, evidências relevantes para a prática clínica da Enfermagem, como ensaios clínicos randomizados, observacionais e metodológicos, mostram que as TE predominantes são baseadas em mensagens de texto, com ênfase no uso de smartphones e internet. A análise dos artigos resultou em duas categorias: TE relacionadas ao comportamento sexual de risco e planejamento reprodutivo, e TE relacionadas ao ciclo gravídico puerperal. Essa abordagem visa subsidiar o cuidado com segurança, eficácia e qualidade na prática clínica de Enfermagem.

### Tecnologias educacionais relacionadas ao comportamento sexual e planejamento reprodutivo

Os protocolos descentralizados de abordagem ao HIV têm fortalecido o acesso à TARV, no entanto, PVHIV tem suas necessidades de saúde integral não atendidas, a exemplo das questões relacionadas a saúde sexual e reprodutiva^(21,24,25)^. O planejamento reprodutivo permite que as mulheres que convivem com HIV/aids tenham escolhas reprodutivas e de comportamento sexual que impactem positivamente na saúde reprodutiva, como, por exemplo, espaçamento entre as gestações e uso de contraceptivos^(23)^.

Estima-se que mais de 80% das gestações não planejadas e/ou indesejadas entre essas mulheres são resultado de necessidades em saúde sexual e reprodutiva não atendidas de maneira eficaz, evidenciando um problema de saúde pública quando relacionado a desfechos desfavoráveis, como prematuridade, início tardio do pré-natal, atraso no acesso e baixa adesão à TARV, aumentando o risco de transmissão vertical^(23)^.

As intervenções encontradas foram eficazes para melhorar a adesão à TARV tanto para as mulheres, quanto para as suas parcerias, por concentrar habilidades e estratégias cognitivo-comportamentais para resolver barreiras individuais, situacionais e estruturais^(20,21)^. Dessa forma, as técnicas de desenvolvimento de competências cognitivo-comportamentais também são fundamentais^(11)^ para as intervenções de saúde sexual mais eficazes.

A análise dos tipos de TE utilizadas para prevenção do comportamento sexual e planejamento reprodutivo de mulheres que convivem com HIV/aids totalizaram quatro artigos^(20-23)^, com pouca divulgação entre os estudos^(20)^. Destes, dois^(20,21)^ eram destinados a PVHIV incluindo mulheres, um em casais sorodiferentes^(22)^, um^(23)^ por especialistas na área e, também, casais sorodiferentes.

Nas publicações analisadas, constatou-se predominância das TE duras, ou seja, das que necessitam de recursos tecnológicos, encontradas em três dos estudos^(20-22)^, voltadas para os seguintes temas: prevenção da transmissão sexual do HIV, planejamento reprodutivo seguro^(20,21)^, uso da TARV pelo parceiro infectado pelo HIV, Profilaxia Pré Exposição (PrEP) pelo parceiro não infectado pelo HIV, sexo sem preservativo restrito aos dias com pico de fertilidade, testes e tratamento para IST, voluntariedade à circuncisão médica masculina, a auto inseminação vaginal e as intervenções reprodutivas assistidas. Todos os estudos refletiram a minimização do comportamento de risco em casais sorodiferentes, ao mesmo tempo que apoiavam os desejos de gravidez.

No entanto, apenas um estudo^(21)^ evidenciou o uso de métodos contraceptivos como uma informação necessária a ser difundida entre as mulheres que convivem com HIV/aids, embora abordado de forma superficial. Isso demonstra a necessidade da ampliação de estratégias que busquem garantir direitos sexuais e reprodutivos, não somente no tocante à concepção segura, mas ao mesmo tempo, orientar sobre as opções contraceptivas disponíveis e efetivas, que contemplem o planejamento reprodutivo, acolhimento e aconselhamento de mulheres convivendo com HIV/aids, com base em evidências científicas.

De forma geral, os resultados mostraram que as TE desenvolvidas reduziram significativamente a carga viral do HIV^(20,22)^, aumentaram a adesão à TARV^(20,21)^ e reduziram novos diagnósticos de IST^(21)^. Além disso, quatro aspectos foram evidenciados positivamente entre os estudos: facilidade de responder às mensagens SMS e incorporar as mensagens no dia a dia^(20,22-28)^; preocupações em torno da confidencialidade, divulgação das informações^(20-23)^; aceitabilidade das ferramentas *mHealth* para ajudar no monitoramento da fertilidade^(20-22)^; e utilidade das ferramentas *mHealth* para melhorar a comunicação entre casais e profissionais de saúde^(21,23)^.

As Tecnologias Educacionais (TE) analisadas incluem aplicativos de mensagens de texto^(20,21,24-28)^, *software* ^(22)^ e cartilhas educacionais^(23)^. Esses métodos, destacando a mHealth, facilitam a participação e envolvimento dos participantes ao tornar a informação sobre prevenção de comportamento sexual de risco e planejamento reprodutivo mais acessível. Um estudo utilizou uma cartilha educacional para direcionar práticas sexuais e reprodutivas seguras a pessoas vivendo com HIV (PVHIV) e seus parceiros, promovendo corresponsabilidade e autonomia na gestão da própria saúde.

### Tecnologias educacionais relacionadas ao ciclo gravídico puerperal

O risco aumentado para a infecção pelo HIV também é referenciado em gestantes, as quais devem ser priorizadas em virtude dos contextos históricos, educacionais e estruturais aos quais estão inseridas^(25)^. Essas condições refletem a presença de obstáculos, barreiras e desafios assistenciais para o enfrentamento e controle da epidemia do HIV/aids, não somente no Brasil, mas no mundo, e podem explicar a alta prevalência da transmissão vertical.

Estima-se que muitas mulheres grávidas soropositivas perdem o acompanhamento após o parto^(24,25)^. Atualmente é recomendado a periodicidade de testes sorológicos de HIV mensal nos primeiros seis meses e, no mínimo, bimestral a partir do 1º ano de vida para mães e bebês expostos ao HIV^(30)^. Essa foi uma das preocupações abordadas nas TE desenvolvidas pelos estudos^(23,24,26)^.

Nesta categoria, seis estudos utilizaram TE relacionadas às mulheres que convivem com HIV/aids durante o ciclo gravídico puerperal^(24-28)^, incluindo intervenções baseadas em mensagens de texto^(24-27)^, e aplicativo^(28)^. Assim, percebe-se um esforço para desenvolver e difundir TE eletrônicas voltadas ao HIV durante o ciclo gravídico puerperal. O despertar para o uso das TE de informação e comunicação é fundamental, pois favoreceu a acessibilidade, a difusão do conhecimento, a atratividade e o acesso rápido à informação mediante os estudos apresentados^(24-28)^.

O desenvolvimento de TE que utilizam um desenho metodológico - como ensaios randomizados^(24-28)^ - foi identificado na análise dos estudos, demonstrando o alto rigor metodológico utilizado no desenvolvimento de TE para as mulheres convivendo com HIV/aids durante o ciclo gravídico puerperal. Tais estudos são essenciais para os sistemas de saúde e para a prática de Enfermagem, devido à sua capacidade de revelar a causa e o efeito de diferentes intervenções^(29,30)^.

Observou-se que o acesso ao telemóvel foi elevado entre os estudos^(24-28)^. Apenas uma pequena parcela das mulheres que participaram dos estudos foi excluída devido à falta de acesso ao telemóvel^(24-28)^. Tal fator coincide com outras evidências disponíveis na literatura, recomendando uma maior utilização das tecnologias disponíveis para melhorar o acesso e a adesão ao acompanhamento nos serviços de saúde e à TARV^(31,32)^. Dessa forma, contribui-se também para o aumento do uso de telecomunicações como parte dos cuidados e criação de vínculo entre o profissional da saúde e a comunidade^(24,33)^.

As TE, enquanto estratégias de saúde pública, quando adaptadas individualmente, podem ser mais eficazes do que intervenções genéricas^(34)^. Um ponto forte retratado em um dos estudos foi a personalização das mensagens de texto com base na idade gestacional do destinatário, nome, horário preferido, idioma desejado, data do parto e nome do bebê. Isso permitiu direcionar as mensagens a indivíduos, uma funcionalidade que acrescentou um “toque pessoal”. Também foi permitido que os participantes enviassem SMS, ligassem ou solicitassem retorno de chamada. Tal interatividade proporcionou uma oportunidade de responder às necessidades e dúvidas dos participantes^(25)^.

Em outro estudo^(27)^, também foi realizada uma abordagem em que os participantes que tinham revelado o seu estado de sorológico ou não tinham o seu próprio telefone podiam optar por receber conteúdo explícito relacionado com o HIV. Esta abordagem oferece um modelo para permitir que os destinatários da intervenção escolham se receberão SMS abertos relacionados com o HIV com base nas suas preferências e situação individuais, ao mesmo tempo que inclui proteções contra a divulgação do estado sorológico.

Outro aspecto destacado foi o aumento do número médio de consultas e acompanhamento de pré-natal e pós-natal^(24,28)^, além disso, o acesso a uma tecnologia disponível e móvel foi associado ao aumento do número de partos vaginais em um dos estudos^(25)^. Com base nesses resultados encorajadores, as intervenções nacionais *mHealth* materna devem ser seriamente consideradas e avaliadas. A realização de uma intervenção para se assemelhar às condições da vida real e a longa duração do acompanhamento, desde a primeira consulta pré-natal até 30 meses após o parto, também foram outros pontos fortes^(27)^ percebidos.

Há evidências crescentes de que as abordagens por mensagens de texto e aplicativos podem melhorar a adesão aos cuidados relacionados ao ciclo gravídico puerperal, mas não há consenso no terreno relativamente ao conteúdo das mensagens de texto, particularmente no grau em que o HIV pode ser abertamente discutido por SMS^(26,35,36)^.

Outra TE para a promoção dos cuidados relacionados ao HIV durante a gestação foi o desenvolvimento de um aplicativo^(28)^ para rastrear a localização das participantes com a finalidade de auxiliá-las na busca por serviços de saúde que ofertem TARV e consultas de pré-natal em caso de viagem. Esse foi um dos primeiros estudos de *mHealth* baseado em GPS, e, embora promissor, enfrentou problemas relacionados à instalação, funcionamento e acesso à internet móvel.

Na Enfermagem, as TE em saúde configuram-se como métodos promissores, atraentes, inovadores e efetivos para direcionar as práticas assistenciais e educativas^(35)^, por favorecer e promover maior adesão ao cuidado contínuo, acolhimento, autocuidado, monitoramento de condições de risco, avaliação de resultados e ampliação de conhecimentos, atitudes e habilidades individuais e coletivas.

Entretanto, as investigações, em sua maioria, concentram-se em identificar o perfil epidemiológico e mensurar os impactos da infecção nos diferentes segmentos sociais^(36)^. Portanto, ainda há lacunas da difusão do conhecimento sobre o desenvolvimento de TE, assim como sobre os seus efeitos no acesso ao cuidado, no comportamento em saúde e na informação da população relacionadas à saúde sexual e HIV/aids.

### Limitações do estudo

A principal limitação desta Revisão Integrativa é a possibilidade de perdas de estudos relevantes indexados em outras bases de dados. No entanto, foi possível mapear estudos expressivos mediante os descritores controlados e suas respectivas sinonímias utilizadas alinhando-os às bases de dados e questão de pesquisa. É importante destacar que vieses de publicação podem estar associados às características específicas de cada instituição onde os estudos incluídos foram conduzidos, impedindo a investigação de abordagens em diferentes contextos na assistência à saúde de mulheres com HIV.

### Contribuições para a Área da Enfermagem e Saúde Pública

Os resultados desta Revisão Integrativa (RI) destacam que a inclusão de estratégias que envolvem tecnologias educacionais é inovadora e tem grande potencial para orientar a prática clínica da enfermagem. O estudo representa um avanço no uso de tecnologias educacionais, essenciais para reduzir sexo desprotegido, promover o uso de preservativos, minimizar comportamentos de risco, diminuir o número de parceiros e aumentar a adesão a testes para HIV, prevenção vertical e cuidados pré e pós-natais. A atuação do enfermeiro é crucial para garantir que essas estratégias sejam compreendidas e aplicadas corretamente, potencializando seus impactos positivos na saúde pública.

Essas estratégias contribuem para o desenvolvimento de conhecimentos e comportamentos relacionados ao planejamento reprodutivo, atendimento, intervenções necessárias, boas práticas e cuidados abrangentes sobre concepção, contracepção e HIV/aids. Nessas áreas, o enfermeiro desempenha um papel fundamental como facilitador da educação em saúde nos serviços de saúde, utilizando seu conhecimento especializado para implementar e adaptar essas tecnologias educacionais de maneira eficaz.

## CONCLUSÕES

Esta revisão proporcionou uma síntese relevante da literatura sobre o desenvolvimento de Tecnologias Educacionais (TE), destacando softwares, aplicativos, mensagens de texto e cartilhas. Os estudos evidenciaram o potencial dessas TE na promoção do conhecimento e comportamento na saúde sexual e no planejamento reprodutivo, validando sua eficácia, sensibilidade e precisão em diversos contextos de atuação da Enfermagem na saúde da mulher. Intervenções por meio de smartphones e mensagens de texto foram predominantes, apresentando notável potencial para expandir a abrangência da *mHealth* ao superar barreiras físicas e mitigar estigmas.

Essas abordagens proporcionam acesso à informação, apoiam a adesão aos cuidados e promovem a autonomia de maneira privativa e consciente, contribuindo para aprimorar o planejamento reprodutivo, a assistência, o direcionamento do atendimento e a formulação de cuidados integrais.
